# Designer TGFβ Superfamily Ligands with Diversified Functionality

**DOI:** 10.1371/journal.pone.0026402

**Published:** 2011-11-04

**Authors:** George P. Allendorph, Jessica D. Read, Yasuhiko Kawakami, Jonathan A. Kelber, Michael J. Isaacs, Senyon Choe

**Affiliations:** 1 Joint Center for Biosciences at Lee Gil Ya Cancer and Diabetes Institute, Gachon University for Medicine and Science, Incheon, Korea; 2 Structural Biology Laboratory Salk Institute for Biology Studies, La Jolla, California, United States of America; 3 Clayton Foundation Laboratories for Peptide Biology, Salk Institute for Biology Studies, La Jolla, California, United States of America; 4 Department of Genetics, Cell Biology and Development, and Stem Cell Institute, University of Minnesota, Minneapolis, Minnesota, United States of America; University of Crete, Greece

## Abstract

Transforming Growth Factor – beta (TGFβ) superfamily ligands, including Activins, Growth and Differentiation Factors (GDFs), and Bone Morphogenetic Proteins (BMPs), are excellent targets for protein-based therapeutics because of their pervasiveness in numerous developmental and cellular processes. We developed a strategy termed RASCH (Random Assembly of Segmental Chimera and Heteromer), to engineer chemically-refoldable TGFβ superfamily ligands with unique signaling properties. One of these engineered ligands, AB208, created from Activin-βA and BMP-2 sequences, exhibits the refolding characteristics of BMP-2 while possessing Activin-like signaling attributes. Further, we find several additional ligands, AB204, AB211, and AB215, which initiate the intracellular Smad1-mediated signaling pathways more strongly than BMP-2 but show no sensitivity to the natural BMP antagonist Noggin unlike natural BMP-2. In another design, incorporation of a short N-terminal segment from BMP-2 was sufficient to enable chemical refolding of BMP-9, without which was never produced nor refolded. Our studies show that the RASCH strategy enables us to expand the functional repertoire of TGFβ superfamily ligands through development of novel chimeric TGFβ ligands with diverse biological and clinical values.

## Introduction

The Transforming Growth Factor-beta (TGFβ) superfamily ligands encompass several subfamilies consisting of TGFβ, Bone Morphogenetic Proteins (BMPs), Activin and Inhibin, Growth and Differentiation Factors (GDFs), Nodal, and Müllerian Inhibiting Substance (MIS). Since TGFβ1, the founding member of the superfamily, was first discovered by Roberts and her colleagues [1], 33 such TGFβ superfamily ligands have been identified in the human genome. These superfamily ligands collectively function in a diverse range of cell types and play important roles in fundamental cellular events including dorsal/ventral patterning and left/right axis determination as well as bone formation and tissue and organ development [2]. By the same token, many TGFβ superfamily ligands are being actively explored for their potential ability to guide the *in vivo* and *ex vivo* differentiation pathways or for the *ex vivo* maintenance of stem cells at various stages [3], [4, and more recent references herein]. Due to their overlapping pervasiveness in their intercellular signaling capabilities, medical intervention of these signaling pathways by TGFβ superfamily ligand holds great promise to develop new treatments of a wide range of different developmental diseases from skeletal and muscle abnormalities to a multitude of neoplastic disorders [5]–[7].

One of the hallmark features of TGFβ superfamily ligands is that they are synthesized as inactive precursor molecules composed of an N-terminal pro-domain and a C-terminal mature domain, which must be cleaved from the pro-domain to become active (e.g. BMP-4) [8]. TGFβ superfamily members are classified based on the conserved structural architecture found in their mature domains. In general, each mature ligand monomer contains 7 cysteines, 6 of which are arranged in the ‘cystine knot’ motif [9]. The last 7th cysteine forms an inter-disulfide bond between two chains, generating a covalently linked dimer. Stretching outward structurally from the centrally located ‘cystine knot’ of the dimer are mainly 4 beta strands forming 2 curved fingers. This gives the dimer the overall appearance of a butterfly. The functional subunit for the TGFβ superfamily can exist both as homo- and hetero-dimers *in vivo* [10]–[13]. Some family members, such as GDF9 and BMP15, lack the cysteine required to form the inter-disulfide bond, yet they are still functional and able to form stable dimers [14].

To initiate the signaling process, TGFβ superfamily dimers must interact with two sets of receptors, termed type I and type II. Both receptors are composed of N-terminal ligand-binding extracelluar domain and C-terminal intracellular serine/threonine kinases. TGFβ superfamily ligands display preferences in their affinity between the two different receptor types. TGFβ, Activin, Nodal, and BMP-7 exhibit high affinity for type II receptors, whereas BMP-2 and GDF-5 possess higher affinity for type I receptors [15]. Following the binding of two high affinity receptors, two lower affinity receptors can then be recruited. Upon binding of all four receptors to the ligand, forming a 6-member ternary complex, the downstream signaling response can be initiated [16]. The constitutively active type II receptors phosphorylate the type I receptors which, in turn, bind and phosphorylate intracellular signaling molecules called Smads. The activated Smad molecules are then able to translocate to the nucleus and interact directly with transcriptional regulators [17], [18]. Therefore, multiple mechanisms can be employed to regulate TGFβ signaling at various stages of the signaling cascade. Extracellular antagonists (e.g. Noggin and Follistatin) by sequestering the ligands from binding their cell surface receptors, cell surface pseudo-receptors lacking the intracellular kinase domain (e.g. BAMBI) by interfering with the phosphorylation step, and intracellular molecules, such as inhibitory Smads by blocking the entry into the nucleus, can play a critical biological role in controlling the signal to reach the nucleus from outside the cell [18], [19].

One of the most intriguing features of the TGFβ superfamily is the high degree of promiscuity displayed by TGFβ superfamily ligands and their receptors. For 33 TGFβ ligands, there are only 12 receptors (7 type I and 5 type II), with which one of each type needs to bind to a single ligand to initiate the signaling cascade. TGFβ superfamily ligands indeed interact with a multitude of different receptors with varying affinity and specificity. For instance, BMP-2 is known to preferentially bind BMPRII, but is also capable of interacting with ActRII and ActRIIb [20]. The structural details of how ligand∶receptor affinity and specificity are regulated and their implications on overall signaling fate are just beginning to be understood. In GDF-5, a single amino acid has been identified for selecting its type I receptor preference [21], while in BMP-3 a single point mutation was discovered which not only alters type II receptor affinity but also endows the ligand with signaling properties [22]. These studies suggest a direct correlation between ligand function and receptor binding and outline a method to altering signaling outcomes through modification of receptor binding patterns.

Although all these TGF-beta superfamily ligands and their sequence variants are of potential therapeutic use, the inability to produce readily significant quantities of pure, active TGFβ superfamily ligands has been a significant barrier to fully explore their clinical potential. Only a limited number of BMP/GDF family ligands have been successfully produced bacterially and refolded to high yields, which include BMP-2, BMP-3, BMP-6, and GDF-5 [21]–[24]. *E. coli* production and the chemical refolding of other ligands, such as Activin βA and BMP-7, have been reported [25], but repeating these results has proven to be difficult. Alternatively, eukaryotic expression systems can be successfully used to obtain certain TGFβ superfamily ligands. Activin-βA has been expressed using stably transfected cell lines, such as CHO cells [26], or transiently transfected cell lines, such as HEK cells [27]. Although these expression systems produce active and posttranslationally-modified TGFβ ligands, they can require long periods of time to establish a production cell line, and be inadequate for screening a large number of sequence variants. The chemical refolding process starting from inclusion body isolated from *E. coli* culture as we used for this study offers a time-effective alternative to produce and screen TGFβ superfamily ligands and their variants.

In the current study, we developed a segmental gene cloning strategy termed ‘Random Assembly of Segmental Chimera and Heteromers (RASCH)’, by which one can create TGFβ superfamily ligands and their chimeric variants with unique characteristics. By applying the RASCH strategy to Activin-βA and BMP-2 sequences, we demonstrate the ability to generate an Activin/BMP-2 (AB2) chimera library, from which we found AB208 with the refolding efficiency of BMP-2 and the signaling properties of Activin-βA. This strategy also produced a significant number of AB2 chimera ligands possessing unique receptor binding and cell signaling properties. Three of these AB2 chimeric ligands, AB204, AB211, and AB215, displayed higher signaling activity compared to BMP-2 and the activity could not be attenuated by the extra- Noggin. In another example, inclusion of a small N-terminal section of BMP-2 was sufficient to develop a functional, efficient refolding of BB29, which resembles the activity of BMP-9. These results provide a groundwork to diversify the biological functions of TGFβ superfamily ligands using the RASCH strategy to expand their clinical potential as novel therapeutic agents.

## Results

### Design Strategy for Chimeric TGFβ family Ligands

The overall RASCH strategy to create our novel TGFβ superfamily ligands is based on a modified directed evolution approach, by which we systematically swap five intact, defined protein segments of two or more TGFβ superfamily ligands instead of random mutagenesis or gene shuffling [28]. This initial approach involved Activin and BMP-2 as two target genes, with which we created, refolded and screened 32 (2^5^) chimeric variants that we refer to as Activin/BMP-2 (AB2) library chimeras. For this experiment, Activin-βA is termed the target ligand because we have not been able to refold despite a report of successful refolding of Activin-βA [25]. BMP-2 is termed the template ligand because we can refold it with excellent efficiency. Typically, >10% BMP-2 dimer product was yielded starting from denatured inclusion bodies, and these dimers have been used in both *in vitro* and *in vivo* experiments and for crystal structure determination [23], [29].

**Figure 1:**
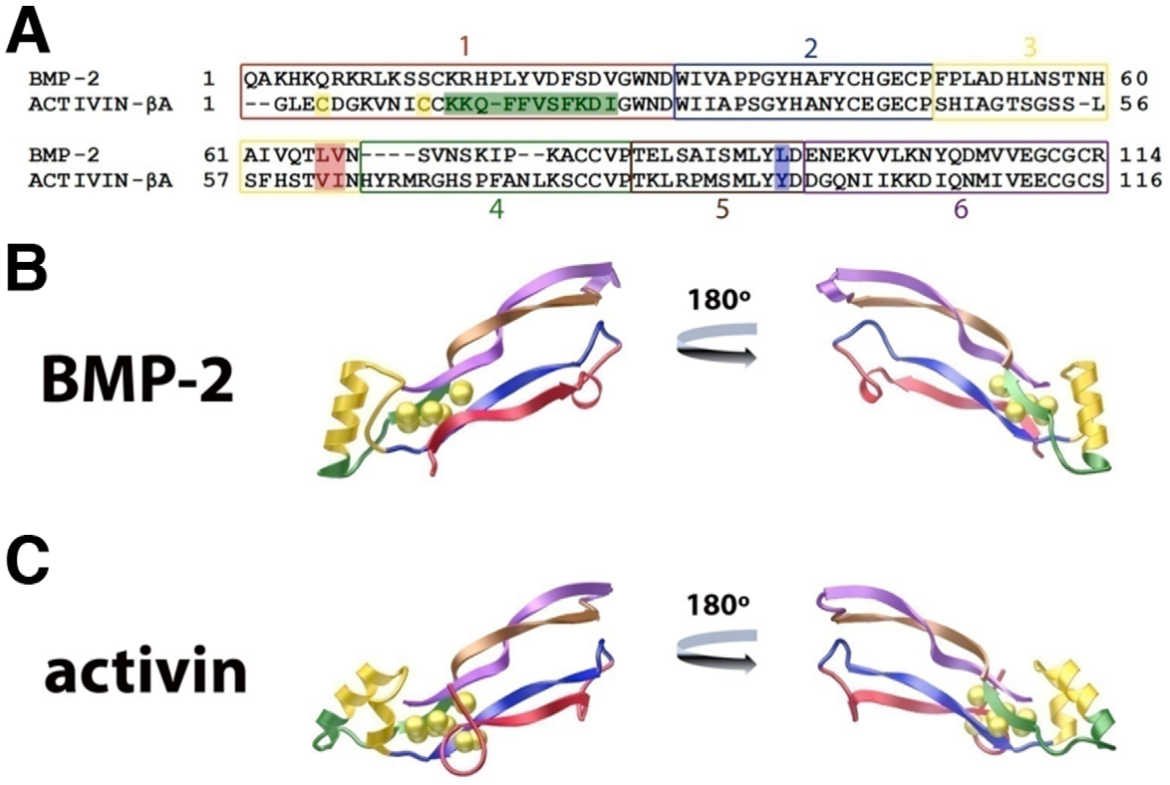
Segmental Design Strategy. Activin/BMP-2 (AB2) chimeras were designed based on structural and sequence considerations. A) Sequence alignment of BMP-2 and Activin-βA with the segments colored and labeled. Yellow boxes indicate Cys residues in Segment 1 of Activin-βA and the Green box highlights the sequence changed to make AB210, while Red and Blue boxes highlight differences in sequence at the boundary regions. How the segments divide the ligands are illustrated in the monomer structure of BMP-2 (B) and Activin-βA (C), with the segment matching the color of sequence box (A). Sulfur atoms of disulfide bonds are represented as yellow spheres.

Sequence boundaries of the five segments of Activin-βA and BMP-2 to create the AB2 chimeras were defined on the basis of their structural elements and the sequence similarity between them. Initially, the crystal structures of Activin-βA [29] and BMP-2 [23] were compared and 6 distinct structural segments were identified to minimize disruption of their secondary structural elements thus to conserve local structural elements as a result of swapping (Figure 1A–C). The exact segment boundaries were further refined following a protein sequence alignment of these ligands (Figure 1A–C), with additional adjustment of segmental boundaries to allow amino acids conserved for segment swapping universally (universal joints) among the entire TGFβ superfamily ligands as seamlessly as possible. More specifically, the pre-helix loop and the majority of the α-helix were combined into Segment 3, while the remainder of the α-helix and the beginning of beta strand 3 were placed into Segment 4 (Figure 1A–C, yellow and green). A few additional point mutations were necessary to create universal joints between certain segments. At the end of Segment 3, TLVN of BMP-2, which corresponds to TVIN of the Activin-βA sequence, was taken as the consensus sequence (Figure 1A, red box). At the end of Segment 5, LYLD of BMP-2 is equivalent to LYYD of Activin-βA (Figure 1A, blue box), for which LYYD was taken as the consensus sequence. Finally, the N-terminus of Activin-βA contains 2 additional cysteines, forming form a 4^th^ intra-disulfide bond, compared to BMP-2 (Figure 1A, yellow boxes). To avoid the potential complication in the refolding process by these extra disulfide bond, Segment 1 of Activin-βA was removed from the final pool of segments, thus it consists of 11 Segments (Segments 1 through 6 of BMP-2 and Segments 2 through 6 of Activin-βA).

### Expression and Refolding of Activin/BMP-2 Chimeras

Each of the 32 AB2 chimera vectors (AB201 through AB232) was assembled individually by PCR from the Activin-βA and BMP-2 segments, and they were expressed using a standard *E. coli* expression system. Surprisingly, all AB2 library chimeras were found in the inclusion body fractions, isolated to high purity and seen as bands around 13 kDa by SDS-PAGE analysis. To standardize the refolding protocol, all AB2 chimeras were refolded in 100 mL volumes at a concentration of 50 mg/L. The concentration was chosen based on successful refolding conditions known for various TGF-beta superfamily ligands including BMP-2, BMP-3, and GDF-5 [23]. The refolding volume was sufficient to ensure that a dimerization yield at the level of 2% or greater results in sufficient quantity to obtain enough protein for biophysical and cell signaling assays, yet the volume still be small enough to be manageable to process the large number of samples simultaneously.

The first evaluation of proper refolding is the formation of dimeric ligand, and all 32 AB2 chimera samples showed the presence of varying degrees of dimer product as determined by non-reduced SDS-PAGE analysis. At this stage, the refolding efficiency was estimated based on the dimerization efficiency, based on which each AB2 chimera was placed into a category ranging from poor (1% or less, −), medium (1–5%), high (5–10%), and highest (>10%, +++) (Table S1). Natural BMP-2 ligand was calibrated as 10% efficiency in comparison. They were classified as a ‘good’ refolder if the refolding efficiency was equal to or greater than 5%. This efficiency of 5% yielding 2.5 mg/L of dimeric protein from a standard 1 L refolding at 50 mg/L would be considered suitable for experiments where large quantities are required. Based on this evaluation, we found that 19 out 32 (∼60%) of the AB2 chimeras met the standard of a ‘good’ refolder (Table S1, ++ or +++).

### Activin-βA Characteristics of AB208 Chimera

**Figure 2:**
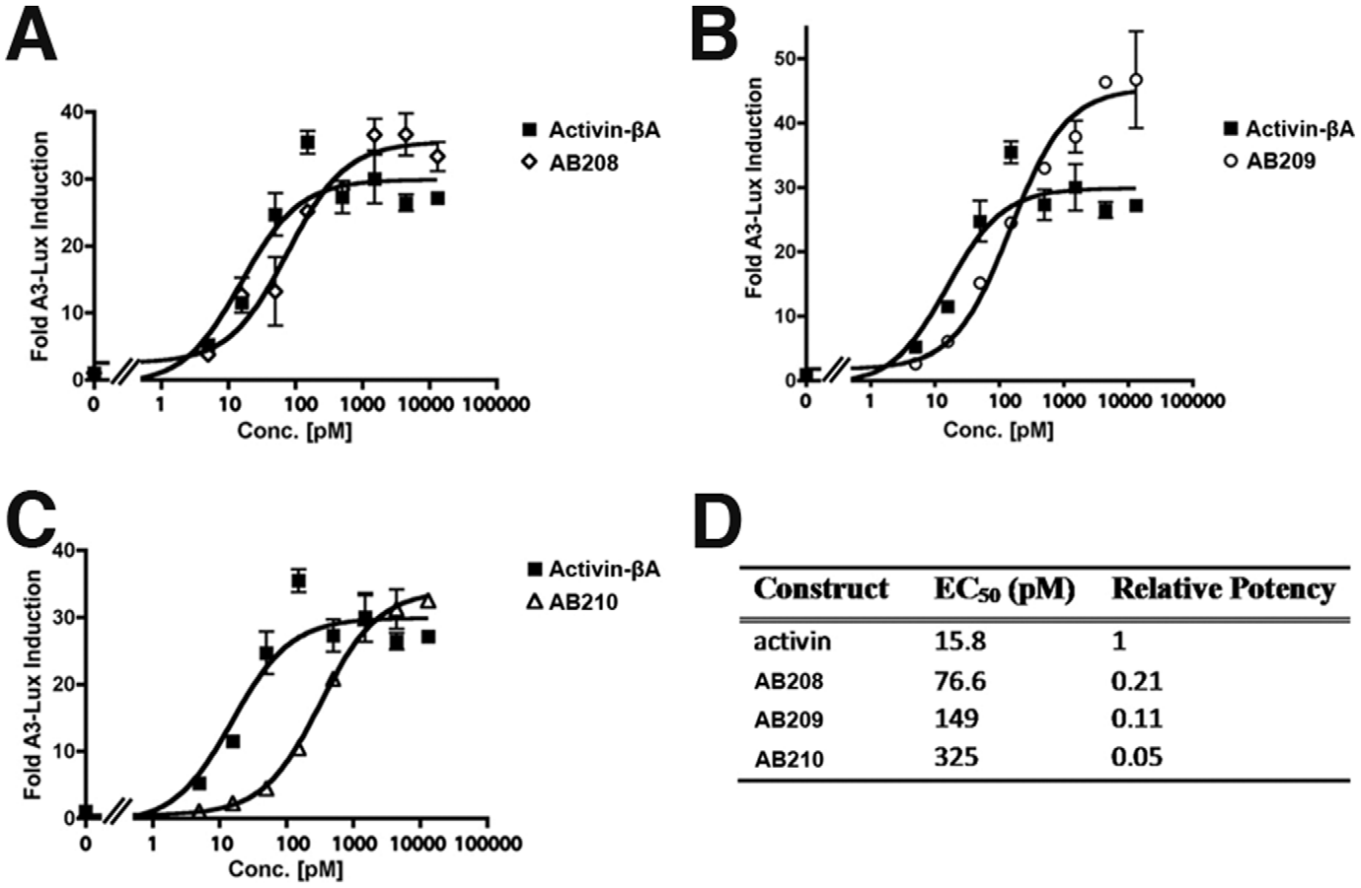
Smad2 Activity Assays. The activity of Activin-βA was compared to (A) AB208, (B) AB209, and (C) AB210 using Smad2 dependent luciferase reporter in HEK293 cells and is presented as Fold Induction. (D) The EC_50_ and relative potency for each ligand are shown. The experiments were performed in triplicate and the error bars are included.

Signaling properties of all AB2 chimeras, regardless of refolding efficiency, were subjected to cell-based activity assays. Activin-βA is known to signal through and activate the Smad2/3 pathway. Using a whole cell luciferase reporter assay sensitive to Smad2 activation, the AB2 chimeras were tested for signaling through Smad2/3 pathway. Out of all AB2 32 chimeras, AB208 is the only one that activated the luciferase reporter in a dose-dependent manner similar to Activin-βA (Figure 2A). AB208 is also referred to by the segmental codename 1b2a3a4a5a6a, which shows that it contains all Activin-βA segments except Segment 1 from BMP-2. Each number in the codename denotes the segment number 1 through 6, and A is for Activin-βA, B is for BMP-2 sequences, and AB2 is a chimera between Activin and BMP-2. We measured that the EC_50_ of AB208 is 76.6 pM. This value is only approximately 5 fold weaker than wild-type Activin-βA (15.8 pM, Figure 2D). Despite its slightly decreased signaling activity, AB208 achieved the same maximal response as Activin-βA (Figure 2A).

To confirm the SMAD2/3 signaling pathway due to Smad2 activation, we tested for the presence of phospho-Smad2. As with Activin-βA, we find that AB208 promotes an increase in phospho-Smad2 levels and not phospho-Smad1 levels (Figure S1). Further, in a Smad1-dependent luciferase assay, AB208 did not exhibit a signaling response, even up to levels of 1 µg/ml. Combined, these results indicate that AB208 specifically activates the Smad2/3 pathway similarly to Activin-βA. We conclude that the designer ligand AB208 exhibits the same signaling functionality like Activin-βA, and it can be produced bacterially from inclusion body with the dimerization yield greater than 10% exceeding that of BMP-2 (Table S1).

### Additional Activin-like Chimeras, AB209 and AB210

Based on the results of AB208, two additional AB chimeras were generated to introduce Activin-βA amino acids back to AB208. AB209 was created by re-introducing the Val-Ile of Activin-βA found at the boundary of segments 3 and 4 into the AB208, where these amino acids of Activin-βA were changed to the corresponding BMP-2 residues Leu-Val due to experimental design constraints (Figure 1A, red boxes). Another chimera, AB210, was created by replacing the C-terminal half of the 1b segment by the equivalent sequence segment of Activin-βA (Figure 1A, green box). This replacement leaves only the 13 N-terminal residues preceding the first structurally conserved Cys-14 (Figure 1A, residues 1–13 BMP-2 numbering), as well as Leu-66, and Val-67 (Figure 1A, BMP-2 numbering) in AB210 as amino acids from BMP-2. AB209 and AB210 were expressed but they were not refolded as efficiently as AB208 (Table S1).

Following refolding and purification, AB209 and AB210 were subjected to the same Smad2 luciferase assay as previously performed with AB208. Functionally, AB209 activated the Smad2 reporter in a dose-dependent manner and displayed activity slightly weaker (less than 2-fold) than AB208 with an EC_50_ of 149 pM (Figure 2B, 2D). AB210 also activated the Smad2 reporter activity with an EC_50_ of 325 pM (Figure 2C, 2D), thus it is ∼4-fold weaker than AB208 or ∼20-fold weaker than Activin-βA (Figure 2D). As with AB208, both AB209 and AB210 showed specific activation of phospho-Smad2 levels (Figure S1). Since AB208's signaling was the closest to Activin-βA in the Smad2-dependent signaling assays, we focused on AB208 for further characterization.

### Receptor Binding Properties of AB208

To address if AB208 signals through and interacts with known Activin-βA receptors similar to Activin-βA, we compared the receptor binding affinities between Activin-βA and AB208 by surface plasmon resonance (Biacore). Activin-βA is known to bind its type II receptors, ActRII and ActRIIb, with high affinity. In our Biacore assay, Activin-βA showed a binding affinity of K_D_ = 0.203 nM against ActRII-ECD immobilized to the chip surface (Table 1). In similar experiments, Activin-βA has been shown to bind ActRII and ActRIIb with sub-nanomolar affinities [29], [30]. When AB208 was passed over the same surface, a binding affinity of K_D_ = 0.344 nM was calculated (Table 1). This result indicates that AB208 exhibits nearly identical type II receptor binding properties as Activin-βA.

**Table 1:** Receptor affinity of BMP-2, Activin A, and AB2 chimeras.

Ligand (Chimera segments)	BMPRIa-ECD		ActRII-ECD	
	k_off_[1/s]/k_on_[1/M*s]	K_D_ [nM]	k_off_[1/s]/k_on_[1/M*s]	K_D_ [nM]
**BMP-2 (BBBBBB)**	**7.54×10^−4^/3.60×10^5^**	**2.09**	**4.05×10^−2^/1.10×10^6^**	**36.8**
**Activin-βA (AAAAAA)**	**No Binding**	**N.A.**	**7.16×10^−4^/3.52×10^6^**	**0.203**
**AB208 (BAAAAA)**	**No Binding**	**N.A.**	**8.24×10^−4^/2.39×10^6^**	**0.344**
**AB204 (BABBAA)**	**1.85×10^−2^/1.13×10^6^**	**16.4**	**1.87×10^−4^/4.91×10^5^**	**0.381**
**AB211 (BBBBAA)**	**1.93×10^−2^/4.07×10^5^**	**47.4**	**3.39×10^−4^/6.46×10^5^**	**0.525**
**AB215 (BABBBA)**	**No Binding**	**N.A.**	**3.71×10^−4^/1.61×10^6^**	**0.230**
**AB212 (BBBBBA)**	**8.48×10^−2^/1.61×10^5^**	**526**	**1.60×10^−3^/3.39×10^6^**	**0.472**

The receptors were immobilized to the chip surface with the ligands flowed over the surface. The data were fit to a kinetic model (1∶1 Langmuir binding with mass transfer) in which K_D_ is calculated as k_off/_k_on_. The table reports data from a single trial. No Binding indicates that the interaction was not detectable.

### Cripto-mediated Signaling Property of AB208

**Figure 3:**
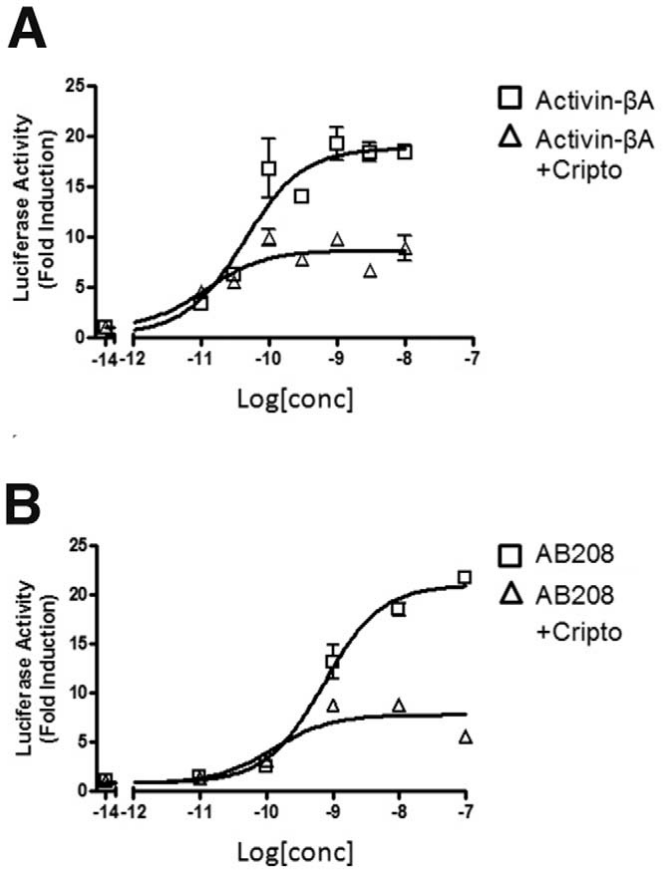
Cripto Binding Experiments. (A) Activin-βA and (B) AB208 activities were tested in the presence and absence of Cripto. The results are shown as Fold Induction of Smad2 luciferase reporter in HEK293 cells. The experiments were performed in triplicate and the error bars are included.

Along with the type I and type II receptors, additional TGFβ modifiers or co-receptors play roles in directing TGFβ ligand signaling. Cripto is one of these TGFβ co-receptors to elicit a diverse range of responses. For instance, the presence of Cripto is required for positive Nodal signaling [31], while it antagonizes Activin-βA [32] signaling. To test if AB208 interacts with Cripto in the same manner as Activin-βA, we measured Smad2 reporter activity in the presence and absence of Cripto. When Cripto was added, Activin-βA signaling was decreased by ∼57% compared to the level achieved by Activin-βA without Cripto (Figure 3A). This reduction in Activin-βA signaling upon the addition of Cripto is consistent with previously reported results [33]. We observe that AB208 signaling was lowered by ∼66% with the addition of Cripto in the same experimental conditions (Figure 3B). Together, the data suggests AB208 possesses the same receptor binding profiles as Activin-βA.

### 1B Segment Improves Refolding of BMP-9

AB208 distinguishes itself from Activin-βA by the presence of the ‘1b’ Segment of BMP-2 and two amino acids (Leu66-Val67). The successful refolding of the AB208 suggested that the ‘1b’ segment contains certain sequence characteristics that may be more universally applicable as a tool to improve the refolding of other currently non-refoldable TGFβ ligands. The ‘1b’ segment comprises the N-terminal residues of BMP-2 forming the first beta strand of finger 1 (Figure 1A, 1B). The analysis of the crystal structure of the ternary complex of BMP-2/BMPRIa-ECD/ActRII-ECD [23] indicates that the majority of the residues found in the ‘1b’ segment do not form contacts with either the type I or type II receptors (data not shown). Therefore, transplanting the 1b segment is unlikely to affect receptor-binding properties of the engineered ligands. We have chosen BMP-9 to test the idea, which exhibits diverse biological functions from involvement in cartilage and bone development to playing a role in glucose regulation [34], [35]. While BMP-9 can be successfully expressed using a CHO cell line [36], BMP-9 has not been refolded *in vitro* from inclusion bodies. A new designer ligand termed 1b_BMP-9 has been created, for which the ‘1b’ Segment of BMP-2 replaced the equivalent segment of BMP-9. 1b_BMP-9 was expressed, refolded, and purified by same methods used for the AB chimeras.

**Figure 4:**
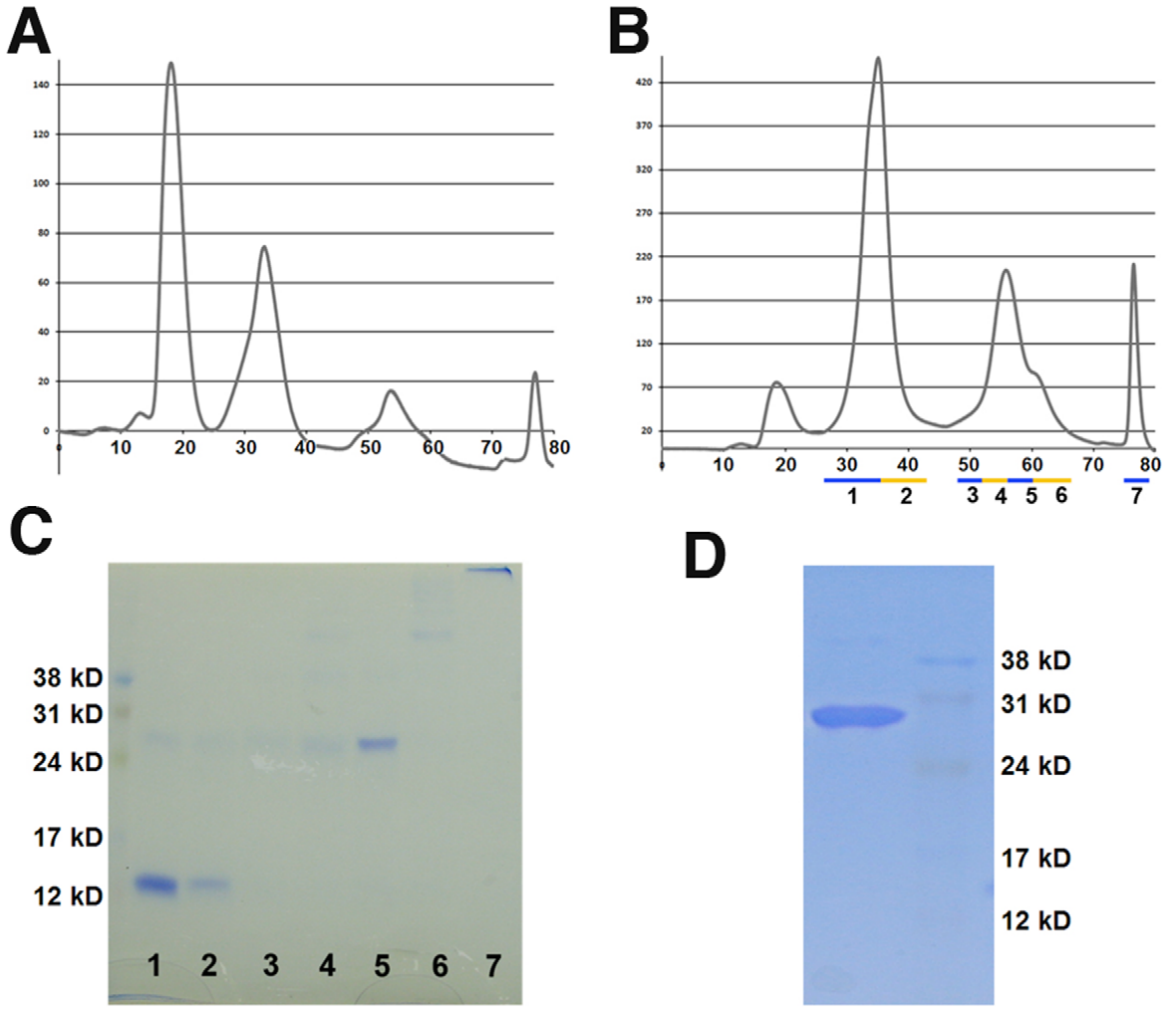
1b_BMP-9 Purification. The refolding properties of BMP-9 and 1b_BMP-9 were compared using standard refolding conditions. The Heparin sulfate chromatography elution profiles are shown for (A) BMP-9 and (B) 1b_BMP-9. (C) The eluted peaks for 1b_BMP-9 were visualized under non-reducing conditions on SDS-PAGE gel. (D) The final product from 1b_BMP-9 refolding and purification is shown by SDS-PAGE gel, using non-reducing conditions.

**Figure 5:**
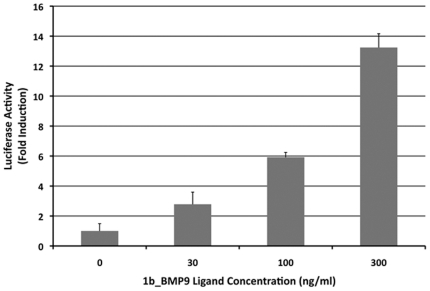
1b_BMP-9 Activity Assay. The activity of 1b_BMP-9 was tested using a Smad1 dependent luciferase reporter in C2C12 cells. The experiment was performed in triplicate and reported as Fold Induction with included error bars.

With respect to the refolding characteristics of 1b_BMP-9, 1b_BMP-9 remains in solution with no visible precipitation, whereas BMP-9 largely precipitates out of solution after 72 hrs in the same refolding condition. With respect to the elution profile from heparin Sulfate purification step, 1b_BMP-9 shows a shift in peaks towards later elution times (Figure 4A, 4B), whereas BMP-9's elution profile displays large peaks early in the run. The total amount of protein available from the refolding step is significantly higher for 1b_BMP-9 than BMP-9. More importantly, when the 1b_BMP-9 elution fractions were run on a SDS-PAGE gel under non-reducing conditions, a band at ∼26 kD, corresponding to the expected dimer size of BMP-9, is mostly present only in fraction 5 (Figure 4C). The area of this peak for 1b_BMP-9 is at least 10-fold larger than the same peak of BMP-9 (Figure 4A, 4B). The overall yield for the 1b_BMP-9 refolding was calculated to be ∼7%, making it be classified as a ‘good’ refolder. Following the reversed-phase chromatography step, the 1b_BMP dimer was purified close to 100% homogeneity (Figure 4D). To test for activity, the purified 1b_BMP-9 was subjected to the same Smad1 luciferase reporter assay. BMP-9 is known to signal through activation of the Smad1 pathway [37]. 1b_BMP-9 shows strong activation of the Smad1 reporter in a dose-dependent manner, indicating that the refolded 1b_BMP-9 is indeed properly folded and functional (Figure 5).

### AB204, AB211, AB212, and AB215 display enhanced BMP-2 properties

**Figure 6:**
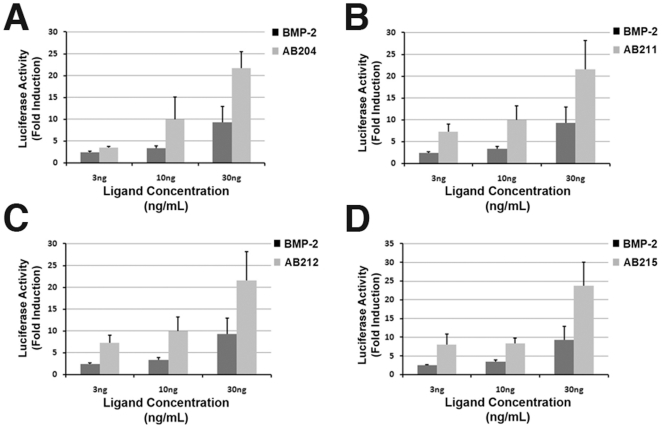
Smad1 Activity Assays. BMP-2 activity was compared to AB2 library chimeras using a Smad1 dependent luciferase reporter in C2C12 cells. Results are shown as Fold Induction for (A) AB204, (B) AB211, (C) AB212, and (D) AB215. The experiments were performed in triplicate and the error bars are included.

Using the classic BMP responsive mouse myoblast C2C12 cell assay with a reporter sensitive to Smad1 activation, we have identified four AB chimera ligands that exhibit the Smad1-mediated signaling properties. The AB2 chimeras AB204, AB211, AB212, and AB215 showed up-regulated signaling activity in the C2C12 cell assays higher than that of BMP-2 (Figure 6A–D). Overall, the chimeras displayed a 3- to 10-fold increase in signaling response compared to BMP-2 with the maximal response at 300 ng/ml for all the ligands.

**Figure 7:**
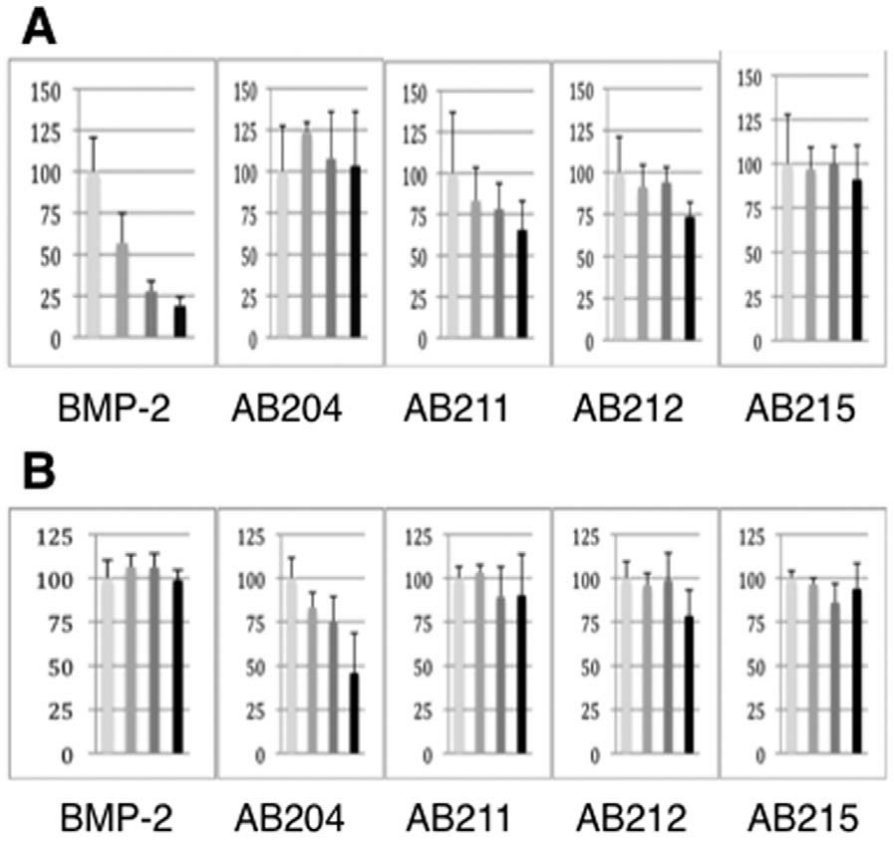
Noggin and Follistatin Inhibition Assays. The ability of Noggin and Follistatin to block ligand-induced signaling was measured using a Smad1 luciferase reporter in C2C12 cells. The ligands are BMP-2, AB204, AB211, AB212, and AB215 from left to right. The activities are shown on relative scale to the control sample (no antagonist treated) at 100% (see Methods). Four columns in each panel are activities tested at 30 ng/ml ligand with and without the presences of 30, 100, and 300 ng/ml (a) Noggin or (b) Follistatin. The experiments were performed in triplicate and reported as Fold Induction with included error bars.

We then checked to see if this increased signaling activity could be attenuated by Noggin like BMP-2. Noggin is an extracellular antagonist for BMP ligands including BMP2, and abolishes BMP-mediated signaling by blocking both receptor-binding sites of the ligand [38]. Unlike BMP-2, Activin-βA signaling is not blocked by Noggin and is instead inhibited by the extracellular antagonist Follistatin [39]. As predicted from the designed structural features of these AB chimeras, they contain segments of the Activin-βA protein sequence and they indeed exhibit different Noggin binding properties as compared to BMP-2. Using the same Smad1-mediated luciferase assay system, BMP-2 activity was significantly blocked to near background levels in the presence of Noggin, whereas the signaling activities of AB204, AB211, and AB215 remained unchanged in the absence or presence of Noggin (Figure 7). Interestingly, the activity of the AB212 chimera is partially blocked to ∼75% of signaling levels by Noggin (Figure 7).

### Receptor Binding Affinities and Specificities of AB204, AB211, AB212, and AB215

The increased signaling activity recorded in the luciferase assays may be a result of the AB2 chimeras possessing unique receptor binding properties compared to BMP-2. AB204, AB211, AB212, and AB215, all share one identical segment, segment 6a derived from Activin-βA (Table S1). Segment 6 comprises beta strand 4 and forms half of the finger 2 region of the ligand (Figure 1B, 1C). This region is known to contain residues important for type II receptor binding for both BMP-2 [23] and Activin-βA [40]. Based on this structural commonality, these AB2 chimeras might have altered affinity for type II receptors compared to that of BMP-2. To test this hypothesis, the binding affinity of the AB2 chimeras to the type II receptor ActRII was measured using Biacore. With ActRII-ECD immobilized to the chip surface, BMP-2 showed medium affinity to ActRII-ECD with a binding affinity of K_D_ = 36.8 nM (Table 1). Activin-βA showed a high binding affinity of K_D_ = 0.203 nM, or ∼180-fold higher than BMP-2 (Table 1). These calculated binding affinities for BMP-2 and Activin-βA are in agreement with our previously data [22], [30]. When the AB2 chimeras were measured against ActRII-ECD, their ActRII-binding affinities were invariably near that of Activin-βA (Table 1). Among them, AB215 possessed the highest affinity of K_D_ = 0.230 nM, with AB204 (K_D_ = 0.381 nM) and AB212 (K_D_ = 0.472 nM) having slightly weaker affinity. AB211 had the weakest affinity with K_D_ = 0.525 nM, which is ∼2.6-fold weaker than Activin-βA (Table 1). These results indicate that these AB2 chimeras possess high affinity for ActRII receptor like Activin-βA.

Since binding to a type I receptor is required for proper signaling, the affinity of these AB2 chimeras for the type I receptor BMPRIa (Alk-3) was also measured. BMPRIa is the preferred type I receptor of BMP-2. When BMPRIa-ECD was immobilized, BMP-2 displayed high affinity with K_D_ = 2.09 nM (Table 1). As expected, no binding was detected when Activin-βA was flown over the same surface (Table 1). Similar to the type II receptor affinity data, these results are consistent with our previous binding experiments [22], [30]. Surprisingly, however, these four AB2 chimeras exhibited varying degrees of BMPRIa receptor-binding affinity compared to BMP-2. AB204 has the highest affinity of the chimeras with an affinity of K_D_ = 16.4 nM, or an 8-fold reduced affinity compared to BMP-2 (Table 1). AB211 shows an even weaker affinity than AB204, with K_D_ = 47.4 nM (∼24-fold lower than BMP-2) (Table 1). Both AB212 and AB215 show significant loss in binding affinity to BMPRIa-ECD. AB212 affinity was calculated to be K_D_ = 526 nM, or a ∼250-fold decrease in affinity, while binding affinity of AB215 to BMPRIa-ECD could not be measured (Table 1). These results implicate that the enhanced SMAD-mediated signaling ability of these designer AB2 chimera does not result simply from the enhanced binding affinity of receptor pairs utilized in nature by BMP-2, but likely from a mix of signaling pathways through different pairs of receptors.

## Discussion

Activin-βA plays important roles in numerous biological functions including follicle and testicular development as well as being implicated in regulation of glucose levels. More recently, Activin-βA has been shown to be instrumental in maintaining pluripotency or ‘stemness’ of cultured human embryonic stem cells (hESCs) [3]. Typically, Activin-βA has been produced in the laboratory either from transiently transfected cell lines [27] or stably transfected cell lines [26], but each of these techniques has its limited use because of a large production cost for a scale-up. Despite a reported method of refolding Activin-βA *in vitro* [25], the method appears difficult to reproduce satisfactorily. Using a segment-swapping RASCH strategy with BMP-2 and Activin-βA, we have demonstrated that a chimeric ligand, AB208 achieved our initial goal to create a new designer molecule that is functionally equivalent to Activin-βA but can be produced relatively easily by chemical refolding. AB208's refolding efficiency is comparable to that of BMP-2 (Table S1) and, in cell signaling assays, it specifically stimulated the Smad2 pathway in the same manner as Activin-βA (Figure 2A; Figure S1). Additionally, AB208 was also shown to bind the type II receptor ActRII (Table 1) and interact with the co-receptor Cripto (Figure 3) equivalently to Activin-βA. In more physiological experiments using cultured rat anterior pituitary cells, addition of either AB208 or Activin-βA induced the release of Follicle Stimulating Hormone (FSH) in a dose-dependent manner (Figure S2). Further, AB208 has been confirmed to maintain the pluripotency of hESCs in defined media just like Activin-βA (unpublished data). Combined together, we conclude that the RASCH strategy indeed led us to create a new designer chimera AB208 that can be used effectively as a replacement for Activin-βA.

It is general that some protein sequences are more readily produced than others by chemical refolding. The exact understanding of why some TGFβ superfamily members refold more efficiently than others remains elusive. For instance, BMP-3 and BMP-6 can be refolded to achieve crystallization-quality samples relatively readily, but BMP-9, BMP-7 or Activin- βA are notably difficult to refold readily [22]. Our study indicates that Segment 1 of BMP-2 (‘1b’) promotes efficient refolding of Activin-βA significantly. When this ‘1b’ segment of BMP-2 was introduced into BMP-9, the refolding efficiency of the new chimera, 1b_BMP-9, was dramatically improved and achieved an overall yield of ∼7%. When tested for functional activity, the refolded 1b_BMP-9 stimulated the Smad1 pathway (Figure 5). Because the yield of recombinant BMP-9 is much lower, we could not obtain sufficient amount of BMP-9 to compare the activity to 1b-BMP-9. So we have not been able to obtain reliable data to compare 1b_BMP-9 to the recombinant BMP-9. There can be other factors noteworthy to the recombinant 1b_BMP-9 and BMP-9. 1b_BMP-9 does not have glycosylation modification. Such difference may result in the difference of the activity in the Smad1-reporter assay when compared to natural mammalian BMP-9. Nonetheless, it is clear that 1b_BMP-9 shows dose-dependent reporter activation, and the 1b modification introduced into 1b_BMP-9 enabled us to overcome the refolding barrier that we encountered with recombinant BMP-9. The properties that allow the ‘1b’ segment to facilitate and improve the refolding characteristics of TGFβ ligands need to be further refined to be universally applicable. For instance, we have not been able to refold 1b_BMP-7. The ‘1b’ segment of BMP-2 contains 7 basic residues preceding the first structurally conserved cysteine, Cys 14 of BMP-2 (Figure 1A). In comparison, the N-terminus of Activin-βA and BMP-9 have only 1 or no basic residues, respectively. Both Arginine and Lysine have been used as additives to induce proper protein refolding through the suppression of protein aggregation [41], [42]. Therefore, the high concentration of these residues in the N-terminus of BMP-2 might function to reduce protein aggregation, thereby promoting refolding. How incorporation of the ‘1b’ segment of BMP-2 further improves refolding of TGF-beta superfamily ligands remains to be better understood.

In addition to refolding specific targets such as Activin-βA and BMP-9, RASCH strategy yielded several AB chimeras with novel signaling properties. Four AB2 library chimeras (AB204, AB211, AB212, and AB215), showed a significant increase in activity compared to BMP-2 in cell signaling assays (Figure 6). The basis for this increased activity appears to be a direct result of their significantly increased binding affinity for the type II receptor ActRII. All four chimeras possess binding affinity for ActRII comparable to Activin-βA, which is ∼180-fold higher than BMP-2's binding affinity (Table 1). Surprisingly, these AB2 chimeras also show varying degrees of reduction in type I receptor-binding affinity to BMPRIa compared to BMP-2 (Table 1). A common sequence region of these four AB2 chimeras is that they all contain segment 6 of Activin-βA (Table S1). Previous structural studies showed that this region is a major site for type II receptor binding [29], [40]. The type II receptor binding site is confined within one subunit of the dimeric ligand, and thus inclusion of segment 6 from Activin-βA would transfer the majority of interface residues into the chimeras. Unlike type II receptor binding, type I receptor binding is composed of residues residing in the pre-helix loop and α-helix of one ligand monomer and residues residing in the inside of the fingers from the other ligand monomer [43]. Therefore, residues in the Activin-βA segments of the chimeras unfavorable towards BMPRIa binding would contribute to both type I receptor binding sites. Unlike type II binding interface, however, defining the segmental origin of residues responsible for specifying type I receptor binding interface is not clear. The structural basis for this reduced affinity is not readily apparent and requires further characterization.

Among the four AB2 chimera, AB215 is peculiar in that it displays high signaling activity despite having no detectable binding to BMPRIa (Figure 6; Table 1). The apparent disconnect between receptor-binding specificity and signaling capability could have two possible explanations. First, the preference for the type I receptor has shifted away from BMPRIa to a different type I receptor including BMPRIb. We have previously shown that a single residue in BMP-3 is responsible for a 30-fold specificity difference for ActRIIb over ActRII [22]. Further, GDF-5's type I receptor preference has also been isolated to a single residue [21]. Therefore, the chimera AB215 may have changed its receptor preference. The C2C12 cells used in our assays are known to express BMPRIa (Alk3) along with BMPRIb (Alk6) and ActRIa (Alk2) and the chimeras could activate the Smad1 reporter using any of these receptors, but we have not been able to evaluate AB215's affinity towards BMPRIb or ActRIa because their ECD reagents are not available for experimental tests. The second possible explanation is that type II receptor binding is the pivotal component for formation of active signaling complexes on the cell surface. The low dissociation rates associated with the tight type II receptor binding would allow the chimeras to bind to the cell surface for long periods of time, effectively increasing their local concentrations to promote binding to type I receptors. Additionally, the receptors are constrained to the membrane surface by their transmembrane domain. So the combination of increased retention time bound to the cell surface with the reduced freedom of the receptors may be sufficient to overcome the decreased type I receptor binding affinity. Even if type I and type II receptors directly interact with each other on the cell surface independent of ligand [44], the reduced affinity of the chimeras for the type I receptor might be overcome by the very high affinity of the type II receptors which bring the type I receptors into the signaling complex.

In addition to having increased signaling activity, AB204, AB211, AB212, and AB215 also possess insensitivity to Noggin. At a Noggin level that completely blocks BMP2 signaling, AB204 and AB215, showed no sensitivity to Noggin, whereas AB211 and AB212's signaling was partially blocked (Figure 7A). For activin-specific antagonist Follistatin, only AB204 showed dose-dependent inhibition of Smad1-mediated signals by Follistatin, whereas other ligands are not significantly affected by Follistatin (Figure 7B). Since AB215 is the only one among four AB2 chimera, which is completely resistant to both Noggin and Follistatin, AB215 appears to be the sequence furthest from either Activin or BMP2 in terms of sequence similarity to Activin or BMP2, while AB204 is closer to Activin and AB211 and AB212 are to BMP2.

The complete insensitivity to Noggin binding appears to require segment 6 of with either segment 2 or segment 5 be replaced (in these cases by those of of Activin-βA). Incorporation of only segment 6 may be partly responsible for Noggin insensitivity displayed by AB212 (Figure 5). Recently, it was reported that the mutation of a single residue in BMP-7 results in a loss in sensitivity to Noggin binding [45]. In GDF-5, a different point mutation has been identified which contributes to Noggin insensitivity [46]. The equivalent residues in our RASCH's segmental design are located in segment 2 and segment 3, respectively, but they differ from each other. Data obtained from these AB2 chimeras are consistent with the notion that the residues responsible for the increased binding affinity to ActRII appear overlapping with those that confer Noggin sensitivity [38].

The conserved architecture found in the mature domain of the TGF-β superfamily ligands allows for the RASCH strategy to be applied to other members of the TGF-β superfamily. Since the same secondary structural features exist in all ligands, their natural functional boundary regions can be swapped to transfer a larger number of receptor binding residues at one time, allowing for the ability to mix different receptor binding properties of a new chimera library. For instance, segment 6 contains the majority of the residues shown to affect type II receptor binding in numerous ligands. One could envision making a designer molecule that binds TGFβRII but uses BMPRIa as the type I receptor, or one could create a Cripto-independent Nodal molecule by mixing segments from a ligand that does not bind Cripto. These unnatural receptor arrangements may stimulate signaling pathways otherwise not naturally activated by any natural ligands in specific tissue type cells. Thus, the ability to engineer novel receptor binding patterns of RASCH-modified (“RASCHonized”) chimeric ligands of TGF-β superfamily may provide a novel platform never existent in nature but conceivably to target them to a specific receptor pair for therapeutic purposes.

## Methods

### Generation of TGFβ Chimeras

To create the AB2 chimera library, the mature domains of human BMP-2 and human Activin-βA were divided into 6 segments (segments 1 through 6) each and primers were designed for each segment. An overlapping PCR strategy was used to mix the various segments together to generate full-length PCR fragment of each chimera. To generate the 1b_BMP-9 chimera, segment 1 of BMP-2 replaced the corresponding N-terminus of BMP-9. Outer primers for all full-length PCR fragments were constructed to incorporate a 5′ *Nde*I site and a 3′ *Xho*I site for cloning into pET21a expression vector. The desired protein sequences were confirmed by DNA sequencing.

### Protein Expression and Purification

All AB2 library chimeras, 1b_BMP-9, and BMP-2 were expressed in *E. coli* as inclusion bodies. The inclusion bodies were isolated, purified, and refolded using a modified protocol [47]. The refolded ligands were purified using Hi-trap Heparin sulfate (GE Healthcare) and reverse phase (GraceVydac) chromatography. Activin-βA was expressed and purified from a stably transfected CHO cell line [48]. Noggin was expressed in *E. coli* as inclusion bodies and purified as previously described [38]. The ECD of human BMPRIa (residues 1–129) was expressed in *E. coli* as a thioredoxin fusion protein using a modification of published procedures [23], [43]. The ECD of mouse ActRII (residues 1–102) was expressed and purified from *Pichia pastoris* as previously described [49].

### Smad1 Luciferase Assays in C2C12 Cells

Smad1-dependent luciferase assays were performed as previously described [50]. In brief, C2C12 myoblast cells [51] are cultured in Dulbecco's minimum essential medium (DMEM)+5% FBS supplemented with L-Glutamine and antibiotics. For luciferase reporter assays, cells were trypsinized, washed twice with PBS and plated into 48-well plates with DMEM+1% FBS. The cells were transfected with −1147Id1-luciferase construct containing the Smad binding sites (Id1-Luc) [50], [52], a Smad1 expression construct, and a reference reporter, pRL-TK plasmid by using Fugene6 (Roche) according to the manufacturer's instruction. Increasing amounts of BMP-2 or the various AB2 chimeras added 24 hours after transfection. Luciferase activity was measured 24 hours after stimulation. The activity of the luciferase reporter was normalized for transfection efficiency by using Renilla luciferase activity, and is reported in fold-induction relative to control values. To test for the ability of Noggin and Follistatin to attenuate the Smad1 signaling of the ligands, the luciferase assays were repeated as described above, with a set dose of Noggin and Follistatin included in the assay. 1b_BMP-9 activity was tested in the same manner as described for the AB2 chimeras.

### Smad2 Luciferase Assays in HEK293 Cells

HEK293T cells [53] were seeded into 24-well plates coated with polylysine at a density of 150,000 cells/well. After 24 h cells were transfected overnight with a mixture of A3 Lux (25 ng) and β-galactosidase (25 ng) reporter plasmids, the transcription factor FAST2 (50 ng), and empty pCDNA3 vector (400 ng) using Perfectin® transfection reagent (GenLantis) according to the manufacturer's recommendations. Then the cells were treated with increasing doses of activin- βA or AB2 chimeras for 16–24 h. The cells were harvested in ice-cold lysis buffer (1% Triton X-100 in 25 mM glycylglycine, 4 nM EGTA, 15 mM MgSO_4_ containing 1 mM dithiothreitol) and assayed for luciferase and β-galactosidase activities using standard methods. To assess the ability of the AB2 chimeras to bind known TGF-β co-receptors, the HEK293T cells were treated with increasing doses of Activin-βA or AB2 chimeras for 16–24 h in the presence or absence of transfected Cripto (mouse Cripto construct was a generous gift from Malcolm Whitman (Department of Cell Biology, Harvard Medical School, Boston, MA). Activity was then measured as previously described [23].

### Surface Plasmon Resonance (BIAcore) Affinity Studies

The affinity of the ligands to the ECDs of BMPRIa and ActRII was measured using a Biacore 2000 (GE Healthcare) and the data were analyzed with BIAevaluation software ver. 4.1 (GE Healthcare). Using primary amine coupling, the receptor ECDs were immobilized on a CM5 chip independently using flow cells 2 and 3, while flow cell 1 was left blank, no immobilized protein, as a negative control. For kinetic analysis, all tests were performed in duplicate using a minimum of five concentrations, plus a zero concentration. The data were fit using a global 1∶1 Langmuir binding with mass transfer model.

## Supporting Information

Figure S1 **Phospho-Smad2 assays.** An increase in phosphor-SMAD1 or phospho-SMAD2 levels were measured in cells treated with different ligands (BMP-2, AB208, AB209, AB210). Unlike BMP-2, these ligands resulted in phosphorylating SMAD2.
(DOC)

Figure S2 **RAPs assay.** Using cultured rat anterior pituitary cells, addition of either AB208, AB209, AB210 or Activin-βA induced the release of Follicle Stimulating Hormone (FSH) in a dose-dependent manner at varying degrees.
(DOC)

Table S1 **Segment contents of AB2 library chimera and their refolding efficiency.**
(DOC)
