# Supplementary figures and images for: Designer TGFβ Superfamily Ligands with Diversified Functionality
Source: PLoS One. 2011 Nov 4;6(11):e26402. doi: 10.1371/journal.pone.0026402 (PMC3208551; doi:10.1371/journal.pone.0026402)

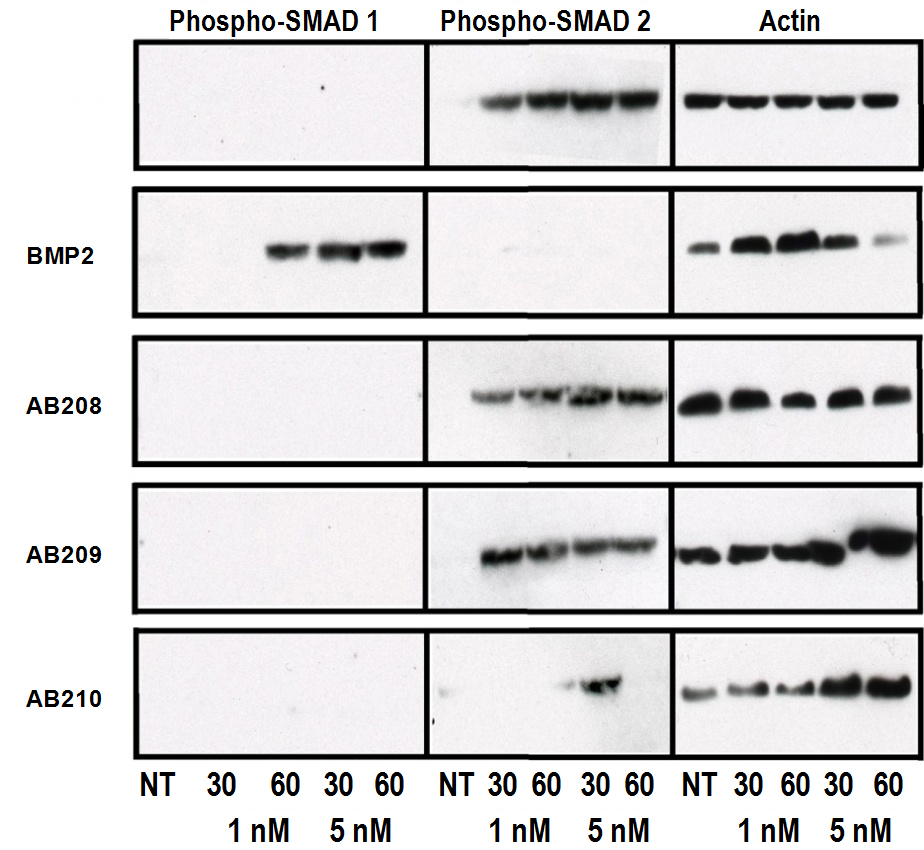


Figure S1: Phospho-Smad2 assays.

Supplement: Figure S1 — Phospho-Smad2 assays. An increase in phosphor-SMAD1 or phospho-SMAD2 levels were measured in cells treated with different ligands (BMP-2, AB208, AB209, AB210). Unlike BMP-2, these ligands resulted in phosphorylating SMAD2. (DOC) [file pone.0026402.s001.doc]

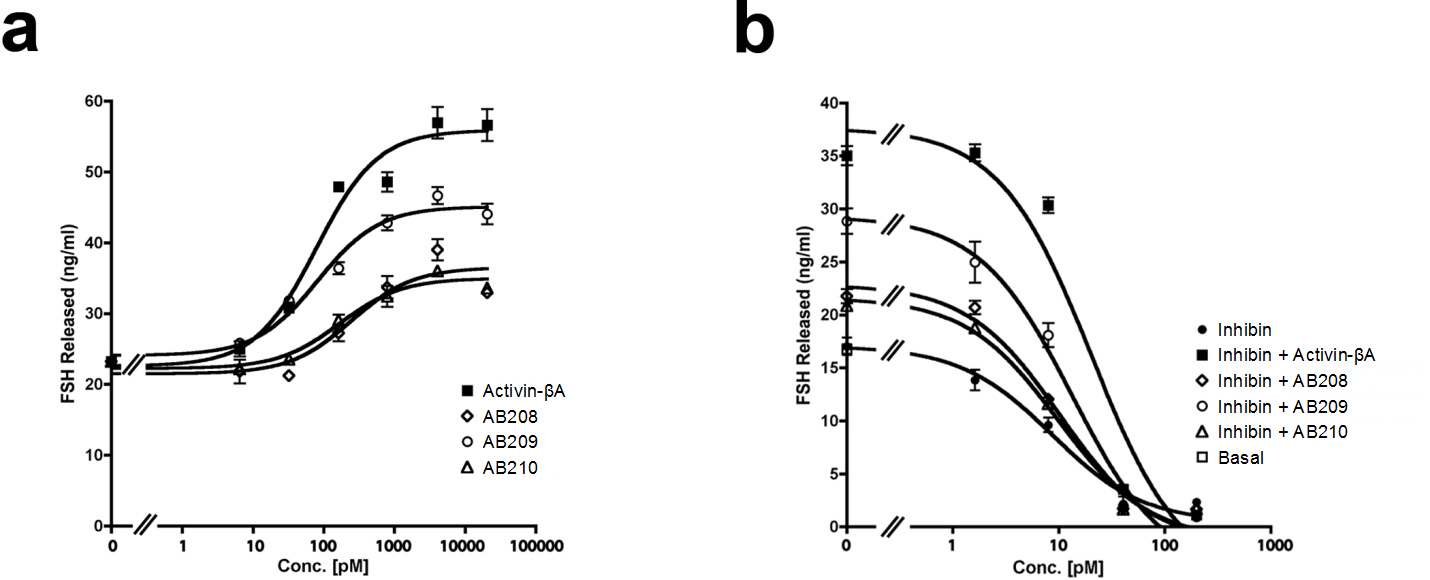


Figure S2: RAPs assay.

Supplement: Figure S2 — RAPs assay. Using cultured rat anterior pituitary cells, addition of either AB208, AB209, AB210 or Activin-βA induced the release of Follicle Stimulating Hormone (FSH) in a dose-dependent manner at varying degrees. (DOC) [file pone.0026402.s002.doc]
