# Supplementary material for: Designer TGFβ Superfamily Ligands with Diversified Functionality
Source: PLoS One. 2011 Nov 4;6(11):e26402. doi: 10.1371/journal.pone.0026402 (PMC3208551; doi:10.1371/journal.pone.0026402)
Supplement: Table S1 — Segment contents of AB2 library chimera and their refolding efficiency. (DOC) [file pone.0026402.s003.doc]

| **Construct (Segment contents)** | **Chimera**  **Name** | **Dimer Yield** | **Yield**  **Rating** |
| --- | --- | --- | --- |
| **1b2b3b4b5b6a (BBBBBA)** | **AB212** | **>10%** | **+++** |
| **1b2b3b4b5a6a (BBBBAA)** | **AB211** | **>10%** | **+++** |
| **1b2b3b4b5a6b (BBBBAB)** | **AB213** | **>10** | **+++** |
| **1b2b3b4a5a6a (BBBAAA)** | **AB217** | **5%** | **+++** |
| **1b2b3b4a5b6b (BBBABB)** | **AB218** | **2%** | **+** |
| **1b2b3b4a5a6b (BBBAAB)** | **AB219** | **3%** | **+** |
| **1b2b3b4a5b6a (BBBABA)** | **AB220** | **3%** | **+** |
| **1b2b3a4a5a6a (BBAAAA)** | **AB201** | **5%** | **++** |
| **1b2b3a4a5a6b (BBAAAB)** | **AB221** | **6%** | **++** |
| **1b2b3a4a5b6b (BBAABB)** | **AB222** | **5%** | **++** |
| **1b2b3a4a5b6a (BBAABA)** | **AB202** | **7%** | **++** |
| **1b2b3a4b5b6b (BBABBB)** | **AB223** | **3%** | **+** |
| **1b2b3a4b5b6a (BBABBA)** | **AB224** | **6%** | **++** |
| **1b2b3a4b5a6a (BBABAA)** | **AB225** | **4%** | **+** |
| **1b2b3a4b5a6b (BBABAB)** | **AB226** | **5%** | **++** |
| **1b2a3a4a5a6a (BAAAAA)** | **AB208** | **>10%** | **+++** |
| **1b2a3a4a5a6b (BAAAAB)** | **AB207** | **>10%** | **+++** |
| **1b2a3a4a5b6b (BAAABB)** | **AB206** | **9%** | **++** |
| **1b2a3a4a5b6a (BAAABA)** | **AB203** | **>10%** | **+++** |
| **1b2a3a4b5b6b (BAABBB)** | **AB227** | **>10%** | **+++** |
| **1b2a3a4b5b6a (BAABBA)** | **AB228** | **4%** | **+** |
| **1b2a3a4b5a6b (BAABAB)** | **AB229** | **1%** | **-** |
| **1b2a3a4b5a6a (BAABAA)** | **AB230** | **2%** | **+** |
| **1b2a3b4b5b6b (BABBBB)** | **AB216** | **>10%** | **+++** |
| **1b2a3b4b5b6a (BABBBA)** | **AB215** | **>10%** | **+++** |
| **1b2a3b4b5a6a (BABBAA)** | **AB204** | **9%** | **++** |
| **1b2a3b4b5a6b (BABBAB)** | **AB214** | **>10%** | **+++** |
| **1b2a3b4a5a6a (BABAAA)** | **AB231** | **4%** | **+** |
| **1b2a3b4a5b6a (BABABA)** | **AB232** | **4%** | **+** |
| **1b2a3b4a5b6b (BABABB)** | **AB233** | **4%** | **+** |
| **1b2a3b4a5a6b (BABAAB)** | **AB234** | **1%** | **-** |
| **1b2a3a4a5a6a (L66V/V67I)**  **(BAAAAA:L66V/V67I)** | **AB209** | **4%** | **+** |
| **1b(1a_II)2a3a4a5a6a**  **(ba-AAAAA)** | **AB210** | **3%** | **+** |

Table S1: Activin/BMP-2 chimera contents and their refolding efficiency
